# Nuclear matrix metalloproteinases: functions resemble the evolution from the intracellular to the extracellular compartment

**DOI:** 10.1038/cddiscovery.2017.36

**Published:** 2017-08-14

**Authors:** Yingqiu Xie, Aidana Mustafa, Adina Yerzhan, Dalmira Merzhakupova, Perizat Yerlan, Askarbek N Orakov, Xiao Wang, Yi Huang, Lixia Miao

**Affiliations:** 1 Department of Biology, School of Science and Technology, Nazarbayev University, Astana, Kazakhstan; 2 College of Basic Medicine, Wuhan University, Wuhan 430071, China; 3 Shandong Analysis and Test Center, Shandong Academy of Sciences, 19 Keyuan Street, Jinan 250014, China; 4 Department of Urology, Shenzhen University Luohu Hospital, Shenzhen Following Precision Medical Research Institute, Luohu Hospital Group, Shenzhen 51800, China

## Abstract

Matrix metalloproteinase (MMP) is defined as an endopeptidase in the extracellular matrix (ECM), which plays essential roles in physiological processes such as organogenesis, wound healing, angiogenesis, apoptosis and motility. MMPs are produced and assembled in the cytoplasm as proenzymes with a cytoplasmic domain and require extracellular activation. MMPs can degrade receptors, extracellular matrix proteins, PARPs and release apoptotic substances. MMPs have been found in the cytosol, organelles and extracellular compartments and recently many types of MMPs have been found in the nucleus. However, the mechanisms and roles of MMPs inside the cell nucleus are still poorly understood. Here we summarized the nuclear localization mechanisms of MMPs and their functions in the nucleus such as apoptosis, tissue remodeling upon injury and cancer progression. Most importantly, we found that nuclear MMPs have evolved to translocate to membrane and target ECM possibly through evolution of nuclear localization signal (NLS), natural selection and anti-apoptotic survival. Thus, the knowledge about the evolution and regulation of nuclear MMPs appears to be essential in understanding a variety of cellular processes along with the development of MMP-targeted therapeutic drugs against the progression of certain diseases.

## Key facts

- Nuclear MMPs are found in disease cases.
- Nuclear localization signal is identified in MMP.
- Some membrane-bound MMPs contain nuclear localization signal.
- Nuclear localization signal is conserved among MMPs, and is comparable to transmembrane domain for conservation among membrane-bound MMPs in different animals.
- Nuclear localization of MMP induces cell death.

## Open questions

- Why is nuclear localization signal conserved among MMPs, and comparable to transmembrane domain for conservation among membrane-bound MMPs in different animals?
- Does it mean that nuclear localization of MMP has an old origin and extracellular localization by transmembrane trafficking is later event during evolution?
- Why does nuclear localization of MMP induce cell death?
- Does it suggest that trafficking from nucleus to membrane is through Darwin’s natural selection based on cell survival?

## Introduction

Matrix metalloproteinases (MMPs) are related to zinc-dependent endopeptidases of Metzicin family.^1^ MMP family has various types of proteins such as MMP-1, MMP-2, MMP-3, MMP-7, MMP-9, MMP-10, MMP-13 and MMP-14. MMPs play essential roles in physiological processes such as organogenesis, tissue wound healing, involution of uterus, angiogenesis, apoptosis, ovulation, cell proliferation and motility.^2^ MMPs are also involved in some abnormal processes including childhood infections, cardiovascular, immune disorders and tumor formation.^3^ In principle, MMPs are essential in intracellular as well as extracellular matrix remodeling.^4^ Some types of MMPs degrade extracellular proteins and extracellular matrix. For example, MMP-3 and MMP-10 can break down fibronectin, proteoglycans and laminin while MMP-8, MMP-13 and MMP-1 degrade collagen types I, II and III.^5^

**Figure 1:**
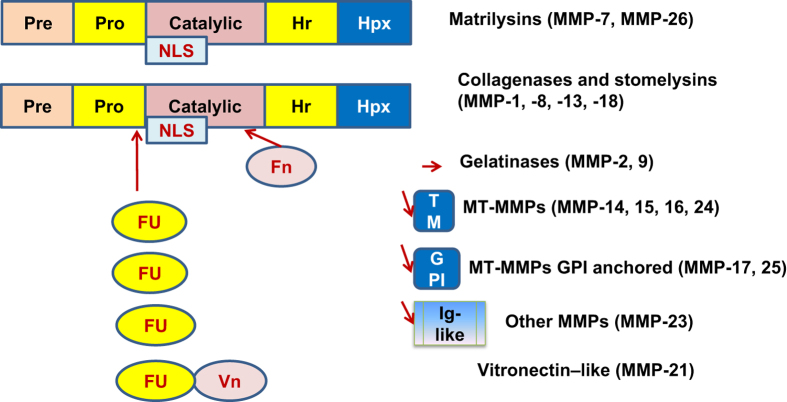
The domain structures of MMP family proteins. All MMPs contain three main domains: pre-domain (Pre), pro-domain (Pro) and the active site or catalytic domain (Cat). Furin recognition motif (Fu) is found between the pro-domain and the active site. Many MMPs possess hemopexin-like (Hpx) region, which is attached to the catalytic domain by a hinge region (Hr). Gelatinases contain gelatin-binding repeats similar to the motif found in fibronectin (Fn) in their catalytic domain. In addition, transmembrane MT-MMPs contain transmembrane (TM) and cytoplasmic (Cy) domains while GPI-linked MT-MMPs contain a glycosylphosphatidylinositol (GPI) portion. MMP-21 contains vitronectin-like domain (Vn) in the catalytic site and MMP-23 contains a unique immunoglobulin-like (Ig-like) domain.

MMP family proteins can be divided into five major groups based on their structural differences: (1) matrilysins; (2) collagenases; (3) gelatinases; (4) transmembrane and GPI-linked MT-MMPs; (5) vitronectin-like and other MMPs.^1^ According to Figure 1, all MMP family members contain a pre-domain sequence that is cleaved while entering endoplasmic reticulum; a pro-domain sequence, which maintains a protein in inactive state and needs to be cleaved for activation, and the active site that is bound to Zn^2+^.^1,2^ Between the pro-domain and the active site there is furin recognition motif, which allows the furin-like proteinases to cleave the pro-domain and activate the zymogen.^1,2^ Many types of MMPs, like MMP-2 and MMP-3 have also been found in the nucleus of the cell.^4,6^ There is strong evidence that MMPs contain nuclear localization signaling sequence (NLS) that allows MMPs to enter the nucleus and regulate certain nuclear events.^1,6^ NLS sequence is mostly found in the catalytic domain and leads to nuclear translocation of these proteins.^6^ The simplest structure described above is possessed by matrilysins, more precisely MMP-7 and MMP-26.^1^ On the other hand, collagenases (MMP-1, MMP-8, MMP-13, MMP-18) contain hemopexin-like domain that is connected to the active site by hinge region.^1^ This domain, which resembles the portion that is made up of four repeated sequences similar to hemopexin and binds to inhibitors of MMPs, is also present in gelatinases (MMP-2, MMP-9), MT-MMPs (MMP-17, MMP-25, MMP-14, MMP-15, MMP-16, MMP-24), vitronectin-like (MMP-21) and other MMPs (MMP-23).^7–9^ Transmembrane MT-MMPs such as MMP-14, MMP-15, MMP-16, MMP-24 also contain a transmembrane domain bound to a cytoplasmic domain, whereas GPI-linked MT-MMPs contain glycosylphosphatidylinositol portion, a glycolipid that allows a protein to attach to the plasma membrane.^3^ MMP-21 contains a vitronectin-like region, a glycoprotein which is the homolog of vitronectin that contributes to cell adhesion and migration.^3^ In the catalytic domain, MMP-23 contains a Ig-like portion bound to hemopexin domain.^2^

It has been shown that MMPs can be regulated at different levels: transcription, activation of zymogen and inhibition of activated forms.^7,8^ In fact, all MMPs are secreted as inactivated enzymes, more precisely in the latent form (zymogens). The activation of MMPs occurs via proteolytic cleavage of their pro-domains,^3^ by the dissociation of zinc ion from cysteine residue.^7,8^ It has been demonstrated that MMPs can be inactivated by tissue inhibitors of metalloproteinase (TIMPs).^9^ The unequal rate of the expression of MMPs and TIMPs can contribute to tumor cell invasion, rheumatoid arthritis, fibrosis and endometriosis.^9^

It was demonstrated that MMPs act as regulatory proteins that cleave and activate several intracellular peptides, such as growth factors and cytokines.^2^ The activation of matrix proteinases causes tissue degradation, fibrosis and matrix destruction.^2^ Further tissue degradation leads to the cancer invasion and the development of secondary tumors, whereas severe fibrosis can be transformed into lung diseases as well as liver cirrhosis.^3^ In addition, MMPs participate in cardiac and vascular remodeling, and their activation can cause the rupture of the atherosclerotic plaque and dilated cardiomyopathy.^10^

MMPs usually localize to the extracellular compartment to cleave the extracellular matrix. However, recent studies suggest that MMPs also localize to the nucleus to play distinct roles.^1^ The present review will summarize the recent progress in studies on nuclear MMPs activation or inhibition via cellular signaling pathways and biological functions.

## Mechanisms of nuclear localization of MMPs

Among MMP subtypes, MMP-2, MMP-3, MMP-9 and MMP-13 have been found inside the nucleus of a variety of cell types of the heart, brain, breast and epithelium.^1^ It has been shown that upon activation, some types of nuclear MMPs are translocated into the nucleus due to the presence of NLS on carboxyl terminus of pro-MMPs.^2^ NLS is a peptide sequence that allows the active transport of a protein to the nucleus of cells.^11^ The composition of the nuclear matrix is similar to the extracellular matrix, though they maintain various cellular functions such as the degradation of nuclear proteins.^12^ Therefore, many MMPs in the nucleus can participate in various nuclear events such as the degradation of nuclear proteins and the regulation of transcription.

We previously compared the putative NLS of different types of MMPs and found that various members of MMP family have conserved NLS.^13^ Particularly, most of them appeared to have in common amino acids of lysine (K), tryptophan (W) and KW sequences. Furthermore, the comparison of NLS of MMP-7 protein in different species established the old evolutionary origin of NLS of MMP-7, which indicates the evolvement of this sequence through evolution.^13^ Other studies showed that deletion of Arg^110^-Lys^111^ and their substitution with Asn and Gln (R110N/K111Q) in NLS of MMP-3 lead to a significant decrease in nuclear localization of MMP-3.^6^ Thus NLS-mediated nuclear localization is one of the mechanisms of nuclear translocation of MMPs. MMPs that do not contain NLS may translocate to the nucleus by cargoes, including RNA, RNA interaction proteins and NLS partner proteins.^14^

In addition, endocytosis may be a mechanism underlying MMPs entering into nucleus.^15^ For example, Membrane Type 1-MMP (MT1-MMP) which is elevated in hepatocellular carcinoma and promotes intrahepatic metastasis interacts and co-localizes with caveolin-1 at the perinuclear compartment.^15^ This finding suggests that MT1-MMP may enter into the nucleus though caveolae by endocytosis.^15^

Moreover, post-translational modifications may affect nuclear localization of MMPs. MMPs may undergo post-translational modifications through Golgi which localizes closely to the nucleus. Recent evidence suggests that MMPs entering into the nucleus might not through accidental invasion via Golgi–ER–nuclear pore. In human endothelial cells, Brefeldin A, an inhibitor that abolishes Golgi endosomal structures did not inhibit MMP-2 nuclear localization, suggesting that nuclear localization of MMP-2 is a stable event independent on the emission of cytosol-nucleus.^4^ In addition, the nuclear MMP-2 was found to be an active form, which has undergone cleavage of pro-enzyme.^4^ Thus, nuclear MMP-2 may have the similar function to extracellular MMP-2 or has been evolved in the nucleus. However, we found that MMP-7 localizes in the nucleus of prostate cancer cells with pro-enzyme form.^16^ These data suggest that MMPs may undergo differential modifications during nuclear translocation. In conclusion, NLS-mediated import may link MMP to Importin proteins^16^ and ARF-mediated protein modification may link MMP to SUMOylation, as we, our collaborators and other labs reported.^17^

## Nuclear MMPs induce apoptosis

Studies showed that MMPs can cleave the nuclear matrix and poly (ADP-ribose) polymerase (PARP), which is one of the components of nuclear matrix.^18,19^ PARP is activated by single-strand DNA breaks induced by peroxynitrite and is responsible for repairing single-strand DNA breaks.^18^ Thus PARP can be inactivated via proteolytic cleavage by MMPs.^18^ Inhibition of DNA repair may result in apoptosis and an excess amount of PARP also may induce the energy depletion of the cells to cause apoptosis.^18^

Studies suggest that MMP-2 can also cleave PARP.^19^ The accumulation of nuclear MMP-2 was first observed in the nucleus of cardiac myocytes.^19^ MMP-2 cleaves PARP to generate two distinct degraded fragments about 66 and 45 kDa, respectively.^19^ Mechanistically, it has been shown that the oxidative stress injury in cardiac myocytes facilitates the synthesis of peroxynitrite, which usually causes DNA strand breaks.^18^ This damage leads to the activation of the PARP.^19^ Thus, the energy deficiency of cells can be avoided by the degradation of the PARP by MMP-2 in the nucleus.^19^ However, nuclear proteolytic cleavage by MMP-2 can be detrimental because this process would inhibit the repair of single-strand DNA breaks.^19^ In addition, nuclear MMP-2 also induces apoptosis in endothelial cells by cigarette smoke exposure.^20^ In details, cigarette smoke stimulates pulmonary artery endothelial cells to generate cleaved PARP and MMP-2 substrate gelatin with increased Annexin V binding in nuclear MMP-2-elevated apoptotic cells, suggesting the role of nuclear MMP-2 in lung alveolar destruction through apoptosis.^20^ Intracellular MMP-2-activated apoptosis was also associated with cleaved PARP, increased Annexin V binding in detached cells for apoptosis of melanoma.^21^

In addition to the nuclear MMP-2, the nuclear MMP-13 also plays essential roles in the apoptosis of neural cells. In general, extracellular MMP-13 breaks down collagens and has been shown to be an important biomarker for breast and other types of cancers.^22–26^ However, after cerebral ischemia, the MMP-13 was found to be localized in the nucleus of neural cells.^22^ This nuclear translocation was associated with oxygen and glucose deprivation of the cells undergoing ischemia.^22^ Although the function of MMP-13 is poorly characterized, it is assumed that the nuclear role of MMP-13 is crucial in inducing the apoptotic cascade due to ischemic stimulus.^22^

In addition, nuclear MMP-3 showed the function of induction of apoptosis. MMP-3 has been found in the nucleus of HepG2 hepatocytes and it has been shown that NLS of MMP-3 localizes in its catalytic domain.^6^ However, nuclear MMP-3 protein was found to be a shorter form of its extracellular protein and one of possible explanations for this can be the cleavage of pro-MMP-3 by intracellular pro-protein convertase furin that may convert it to the active nuclear form.^6^ The nuclear function of MMP-3 is found to be correlated with the induction of apoptosis, based on the evidence that apoptotic cells overexpress MMP-3 in comparison with normal cells.^6^ MMP-3 can induce the cleavage of nuclear proteins such as PARP, and other proteins that participate in DNA repair and mRNA processing.^6^ This can cause a rise in the amount of DNA damage, which will eventually induce apoptosis.^6^

Nuclear MMP-9 also induces apoptosis. Although the function of MMP-9 is distinct in various nuclei of the amygdala,^27^ upregulation of MMP-9 in the nuclei of human atrophic renal tubular cells is associated with human renal fibrosis, which is the formation of scar in the renal tissues.^28^ The exact mechanism that explains these findings is currently unknown. However, it has been shown that the degradation of nuclear proteins, such as PARP and XRCC1, by MMP-9 results in apoptosis and formation of scar tissue.^28^

## Chromosomal (nuclear) MMPs regulate chromosome stability

Nuclear MT1-MMP was found in the centrosome and cleaves centrosomal protein and pericentrin, which are required for centrosome function and mitotic spindle formation. Overexpression of MT1-MMP promotes spindle abnormality, aneuploidy, chromosome instability and thereby tumorigenesis.^29,30^ In details, MT1-MMP can transform human mammary epithelial cells and cleave pericentrin through upregulating oncogenes, such as aurora kinase and *β*-catenin.^30^ Moreover, MT2-MMP (also named as MMP-15), was examined to be localized to chromosome 16q12.1 closing to 16q heterochromatin using FISH *in situ* hybridization analysis, suggesting the role of chromosomal nuclear MMPs in genomic stability.^31^

## Nuclear MMPs in cell migration

Studies have shown that overexpression of MMP-13 plays a role in the metastasis of colon cancer, breast and other cancer types.^24–27,31–33^ For instance, disruption of basement membranes of tissues by upregulated MMP-13 leads to cell dissociation, tumor invasion and metastasis in breast cancer.^33^ MMP-10 was known to induce cell invasion in cervical tumors. However, a new function was found in most recent studies done by Hino *et al*.^34^ that transforming growth factor –*β*1 induces oral squamous cell carcinoma through MMP-10. Studies have shown that MMP-7 is capable of proteolysis, and plays a role in cell dissociation by destroying the tight junctions and degrading collagen type IV that is the skeleton of the basement membranes of blood vessels and a component of the ECM, disruption of the tumor stroma and current vessels, cessation of neovascularization, which eventually lead to necrotic lesions and hypovascularity in tumors and induces invasion of cancer cells and tumor metastasis.^35–37^ Studies by our collaborators showed that MMP-7 nuclear localization induces more aggressive migration.^16^ In detail, nuclear MMP-7 interacts with ARF, an upstream regulator of MDM2–p53–p21 pathway, which facilitates the cellular senescence.^38^ ARF-mediated stabilization of p53 is through disruption of MDM2, an E3 ubiquitin ligase for p53.^38^ Thus p53-dependent conical ARF pathway has long been believed to be related to ageing and tumor suppression. Recent studies have shown that the tumorigenesis role of ARF in cancer independent of p53.^16^ Under PTEN/p53 double null or mutation condition, ARF may exert p53-independent function. We and our collaborators found that MMP-7 nuclear translocation and stability requires ARF-mediated tumor microenvironment for cell migration by disruption of cell adhesions.^16^ Co-overexpression of MMP-7 and ARF resulted in greater cell migration, whereas knockdown of MMP-7 decreased wound healing ability of cells in prostate cancer.^16^ Thus nuclear MMPs may promote cell migration greater than extracellular MMPs.

## Nuclear MMPs in tumorigenesis

It has been shown that overexpression of nuclear MMPs is related to the cancer development.^39^ For instance, cells incapable of apoptosis can induce tumorigenesis. Nuclear MMP-3 is associated with tissue remodeling and cancer progression.^39^ In addition, constant expression of MMP-3 may lead to severe damage of nuclear matrix and the formation of genetic abnormalities.^39^ In recent studies, Sun *et al*.^39^ reported that Bmi-1 induces expression of MMP-3 through NF-*κ*B signaling pathway, which has implication in the progression of glioma.

In prostate cancer, nuclear MMP-7 cooperates with ARF to degrade E-cadherin and extracellular matrix.^16^ Increase in ARF expression is usually linked to prostate cancer progression that is in turn linked to the deletion of PTEN.^16^ We and our collaborators found nuclear MMP-7 involved in PTEN loss-induced tumorigenesis in prostate.^16^ Nuclear MMP-7 expression was frequently seen in invasive front of prostate tumors of Pten/Trp53 knockout mice.^16^ We and our collaborators also found that nuclear-localized MMP-7 is expressed in ARF-associated aggressive human prostate cancer in clinical specimens.^16^ Thus cancer microenvironment may be remolded by nuclear MMP signaling. Originally, extracellular MMP-7 is engaged in the disintegration of Fas ligand, which induces cell invasion that has an anti-apoptotic effect.^40^ Moreover, studies have shown that MMP-7 reduces the ability of natural killer (NK) cells, a part of the immune system, to recognize cancer cells, so the immune system cannot kill tumor cells.^3^ Recent study conducted by Zeng *et al*.^41^ showed that the level of S1P (sphingosine-1-phosphate) induces significant elevation of MMP-7 in hepatocellular carcinoma metastasis. In details, S1P promotes expression and secretion of TGF-*β*1, for feedback loop of shedding of syndecan-1.^41^

Besides nuclear MMP-7, nuclear MT1-MMP plays essential roles in cancer patient survival.^15^ One cohort study examined 101 pairs of hepatocellular carcinoma (HCC) and their adjacent liver tissues, with eight normal liver tissues as control to compare MT1-MMP expression by immunohistochemical analysis.^15^ The authors found that nuclear MT1-MMP expressing patients have worse overall survival rate with more chance to bear big tumor.^15^ Moreover, nuclear MMP-1 expression was detected in the stromal cells of breast cancer. One cohort study without distinguishing nuclear or extracellular MMP-1, showed that total MMP-1 expression in breast cancer patients were correlated with worse survival.^42^ Thus, nuclear MMPs may be associated with aggressive cancer progression and poor survival rate.

## Nuclear MMPs in muscle regeneration after injury

MMPs have essential roles in muscular regeneration after injury by breaking down ECM,^43^ which is significance of clinical surgery. MMP with plasmin is needed during wound healing and plays a role in the adaptation of muscle to trainings.^44^ In addition, MMP in cooperation with urokinase-type plasminogen activator (uPA) and plasmin may trigger some growth factors signaling including basic fibroblast growth factor, which participates in the migration of cells and in the reorganization of tissues.^45^ Furthermore, MMP regulates migration of myoblast to injured area.^45^ Specifically, MMP-9 is assumed to be an activator of satellite cells and MMP-7 has a function in integration of myoblasts.^45^ Moreover, there is evidence that S-phase nuclei-localized MMP-9 regulates cell cycle during differentiation of muscle cells.^45^ In summary, though the detailed mechanisms are largely unknown, recent studies suggest that in addition to extracellular MMPs, nuclear MMPs also play an essential roles in tissue remodeling for organ repair, which is significant in clinical surgery through regulation of nuclear MMPs signaling.

## Nuclear MMPs in ischemic stroke

Nuclear MMPs were discovered to induce neurodegeneration by activating neuronal apoptosis, neuroinflammation, oxidative DNA damage and disrupting blood-brain barrier. Oxidative stress induces nuclear translocation of MMP-2 and MMP-9.^14^ Nuclear MMP-2- or MMP-9-mediated degradation of PARP-1 and XRCC1 results in neuronal accumulation of oxidative DNA in early stages of ischemia.^46^ These proteins are important in base excision repair pathways for DNA repair, cell survival and apoptosis.^14^ As a result, injection of MMP inhibitors represses apoptosis in brain infarction.^46^

## Nuclear MMPs as transcriptional cofactors

It has been shown that extracellular MMPs can regulate transcription through signaling pathways. Particularly, membrane-bound membrane type 1 (MT1)-MMP (also named as MMP-14) can regulate transcription of VEGFA through Src kinase signaling,^47^ Smad1^48^ and Dickkopf-3.^49,50^ It is possible that ECM degradation by MMPs can affect the integrin–ECM interactions, which can produce signals that regulate transcription.^47^ However, whether nuclear MMP-mediated transcriptional regulation is direct has been unclear until recent studies showed that MT1-MMP or MMP in the nucleus can directly regulate transcription.^47–52^

It has been shown that MMP-12 can directly bind to promoter and regulates transcription in immune response.^51,52^ Interferons are proteins that signal the cell on the presence of bacteria, viruses or tumor cells, and induce immune system of the host cells.^51^ After viral infection, macrophages release MMP-12 which is internalized and translocated into the nucleus.^52^ Intracellular MMP-12 binds to NFKIBA promoter region and induces transcription of interferon-alpha (IFN-*α*), so that it can induce antiviral immunity by destroying viral proteins.^51,52^ However, extracellular MMP-12 has shown to degrade IFN-*α* receptor 2 binding site of systemic IFN-*α*, to reduce systemic toxicity.^51,52^ Hence, it was suggested that inhibiting extracellular MMP-12 might lead to new avenues in the development of immune system against pathogenic viruses.^51,52^

In addition, nuclear MMP-3 can bind directly to promoters and regulate transcription of multiple target genes. First, nuclear MMP-3 was identified as a DNA-binding protein in a screening using transcription enhancer dominant in chondrocytes.^53^ Further nuclear MMP-3 was found to bind and transactivate promoter of connective tissue growth factor (CCN2/CTGF).^53^ CCNs regulate wound healing, fibrotic disease, arthritis and cancer; and CCN2 promotes chondrocytic growth.^53^ Further, nuclear MMP-3 can bind heterochromatin protein gamma as cofactors on the promoter of CCN2/CTGF.^53^ Moreover, when we were revising the manuscript, one report just published the most recent finding that nuclear MMP-3 target genes by transcriptional regulation include Heat Shock family proteins such as Hsp70B and Hsp40.^54^ In more details, nuclear MMP-3 can recruit nuclear heat shock factor 1 as a cofactor and bind together to transactivate Hsp70B enhancer.^54^ Mechanistically, hemopexin-like domain is required for nuclear MMP-3 transcriptional activity.^54^

## The evolutionary path of MMPs

MMP proteins are present not only in vertebrates but also in plants and invertebrates, which shows evolutionary history of these proteinase enzymes.^55^ Due to enormous functions of membrane MMPs in comparison with nuclear MMP, the nuclear MMP family might have evolved first. This can be confirmed from figures generated using database and software of phylogenetic trees (Supplementary Data) that compare NLS and TM of MT-MMPs,^56–58^ such as MMP-14 and MMP-15. The resulting similarities between nuclear localization sequences in various species indicate the old evolutionary origin of NLS of this protein family. The comparison of NLS and TM of TM-MMPs can reflect the domain evolution of MMP translocation in different cellular compartment. The surprising results suggest that NLS and TM underwent a parallel evolution and in some species NLS has fewer branches than TM (Supplementary Data). Our analysis suggests that TM-MMPs may have undergone accelerated evolution in TM domain to overcome nuclear localization. Thus nuclear functions of MMP may be ancestral events that evade cancer or severe fatal diseases.

Mutations could lead to the modification of MMP proteins including NLS sequence. Therefore, nuclear MMPs might have been evolved to translocate to the membrane through death-induced natural selection for survival. At that time, both nuclear and membrane MMPs were common, but eventually, as 'only the fittest survives', the function of nuclear MMP that underwent cell death became very limited and the fittest MMPs, membrane MMPs with plenty of functions that enhance cell survival became the most popular type.

Nuclear MMPs, but not extracellular MMPs, in most cases, could induce cell death (apoptosis),^1,6^ because evolution needs clearance of nuclear MMP to allow MMPs to translocate to the membrane. Therefore, cells with nuclear MMPs will eventually die and those cells without expression of nuclear MMPs are more likely to survive. We mentioned above that MMP-2 may contribute to tumor cell apoptosis.^21^ However, it has been shown that MMP-2 co-localizes with TIMP-1 in neonatal rat neurons, while TIMP-1 inhibits MMP-2 function.^21^ Thus, TIMP-1 can prevent MMP-2-induced apoptosis and allow cells without expression of nuclear MMP-2 can survive and evolve. Therefore, TIMP-1 might be the promoter for accelerated evolution of MMPs from nucleus to extracellular compartment. In addition, direct evidence suggests that extracellular MMP-9 and MMP-10 can disrupt apoptosis induced by p53 by multiple mechanisms including survival signaling such as IGF1.^59^ Moreover, nuclear MMP-1 showed anti-apoptosis function by prevention of lamin A/C degradation and inhibition of caspase activation in glial Müller cells.^60^ The counteract of extracellular and nuclear MMPs in inducing and inhibiting apoptosis suggests that the origin of MMPs might be in the nucleus. Apoptosis might be a selective tool to allow MMPs to evolve to localize to extracellular compartment. During the evolution, the nuclear MMP functions might be inhibited by TIMPs or other membrane MMPs for accelerated evolution.

**Figure 2:**
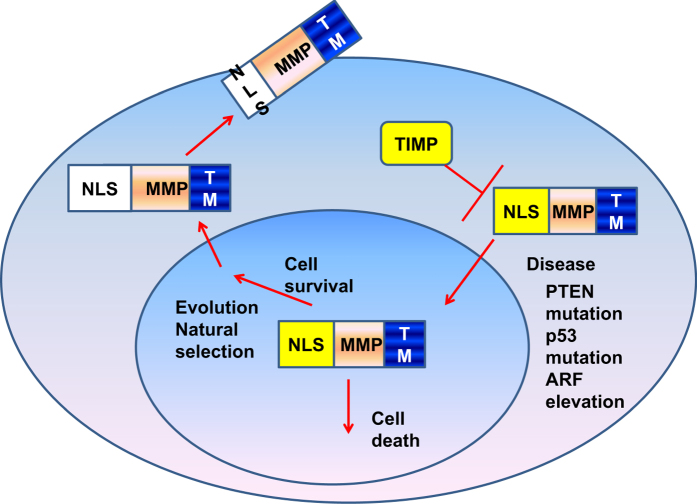
The hypothetical evolutionary path of MMP. Original MMPs may contain strong functional NLS to stabilize MMP in the nucleus through ARF. During evolution, many factors may inhibit MMPs translocation in nucleus such as tissue inhibitors of MMPs (TIMPs) or apoptosis in cells which express nucleus MMPs. Natural selection may allow MMPs to undergo evolution through mutation of NLS to prevent nuclear localization of MMPs. In human diseases induced by mutations such as p53, or PTEN, nuclear MMPs may recover their original character but may gain new functions to induce aggressive disease such as cancer.

Cells that can survive the natural selection by apoptosis induced by nuclear MMPs may have undergone second gene mutation, abnormal signaling elevation or cross-talk signaling complementation. This might lead to cells to be survived, express nuclear MMPs and promote more aggressive diseases. For example, we and our collaborators found that nuclear MMP-7 associates with advanced prostate cancer.^14^ Therefore, the natural selection and fitted survival might give the selection pressure for accelerated evolution of MMPs. In summary, based on our hypothesis, the evolution of MMPs can be outlined as shown in Figure 2.

## Perspectives on mechanisms of nuclear MMP-induced apoptosis

Due to limited literatures for references, the mechanisms underlying the nuclear MMP-induced apoptosis is still unclear. The possibility that nuclear MMPs directly cleave nuclear PARP and caspase is widely accepted because it has been found that the cleaved PARP or caspase associates with nuclear MMPs.^61^ Moreover, other potential nuclear targets of nuclear MMPs associated with apoptosis have been identified as Ste20-like kinases, p21-activated protein kinase, initiator nuclear caspases such as caspase2, caspase 8, caspase 9 and caspase 10.^61^

In addition, reactive oxygen species (ROS) produced by oxidative stress may damage DNA and cause apoptosis or genomic instability. MMP-3 has been shown to induce mitochondrial production of ROS, genomic instability, associate with decreased cell adhesions and oncogenic cellular stress.^37^ The MMP-3-activated ROS may not only damage DNA but also induce apoptosis. However, nuclear MMP-3 can induce apoptosis through the cleavage of nuclear caspase.^61^ Moreover, nuclear MMPs may mediate damaged DNA repair on neuronal death associated with neuroinflammation.^62^ One pathway is through proapoptotic signaling upstream of caspases, such as IL-1*β* signaling.^62^

## Conclusion

**Figure 3:**
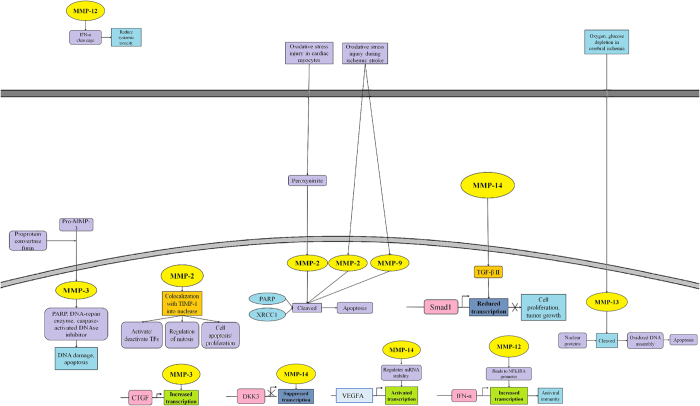
The nuclear functions of MMPs. Nuclear MMPs may have differential functions in different tissues and diseases. Nuclear MMPs can function as a transcription factor through binding to DNA motif in target gene promoters. Nuclear MMP-induced apoptosis in nucleus is common. For details, please refer to references 1–62.

By concluding the review of nuclear MMP family proteinases, many studies have shed the light to both cell death and evolutionary functions. Further research of detailed analysis of structure and function of matrixin is needed to screen inhibitors of proteinases to regulate abnormal cell proliferation, apoptosis or any other toxic effects. Understanding roles of nuclear MMP in signaling cascade (Figure 3) and evolution (Supplementary Data) will create the development of therapeutic avenues. Moreover, gain of knowledge in evolutionary translocation of nuclear MMP to the membrane by cleavage and loss of NLS function in trafficking might shed the light to unknown properties of intracellular evolutionary functions of secreted proteins.
