# Supplementary figures and images for: Nuclear matrix metalloproteinases: functions resemble the evolution from the intracellular to the extracellular compartment
Source: Cell Death Discov. 2017 Aug 14;3:17036–. doi: 10.1038/cddiscovery.2017.36 (PMC5554797; doi:10.1038/cddiscovery.2017.36)

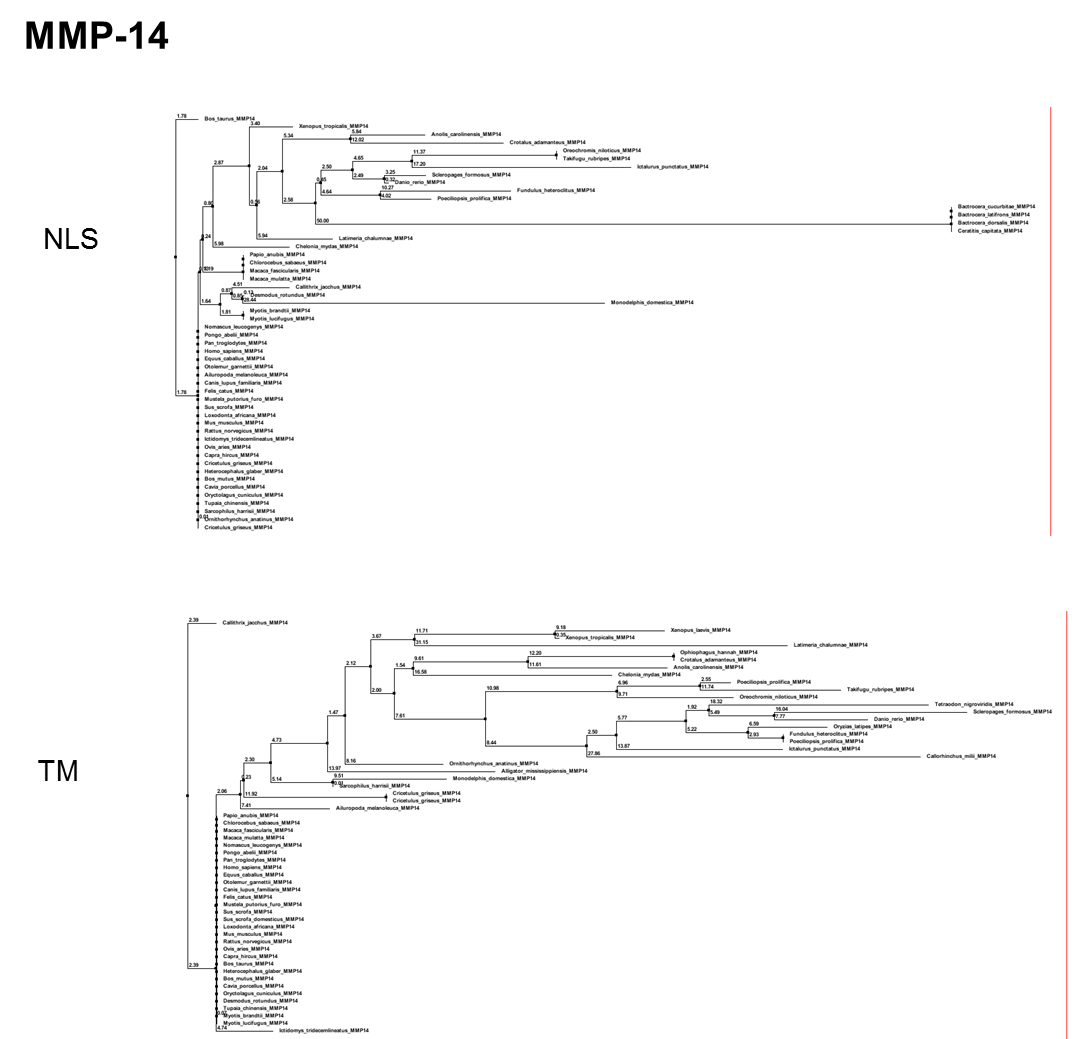

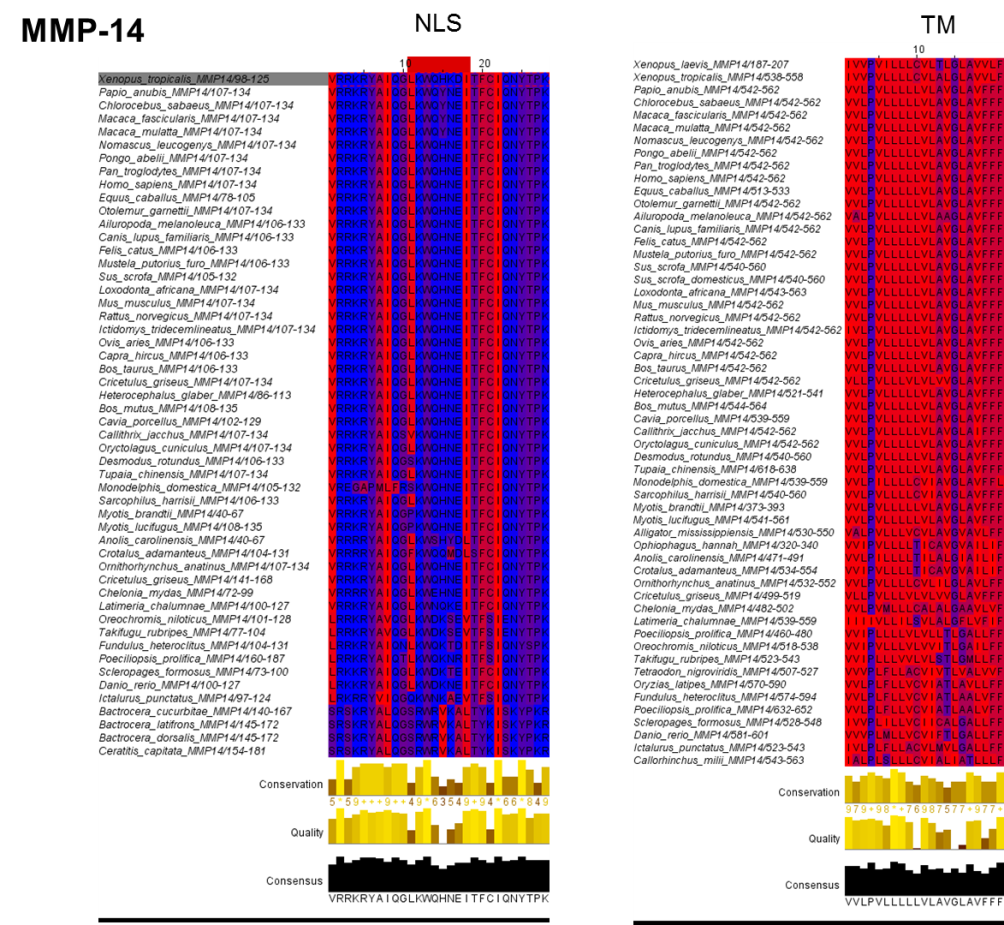


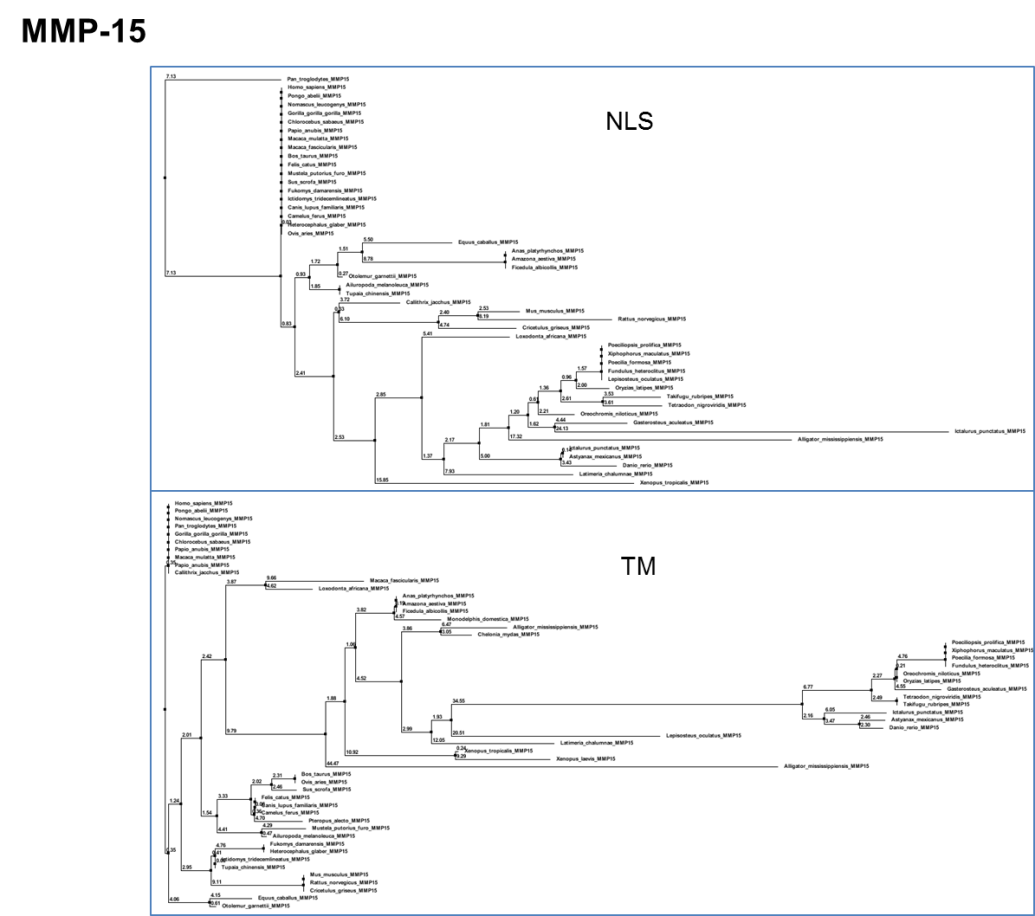


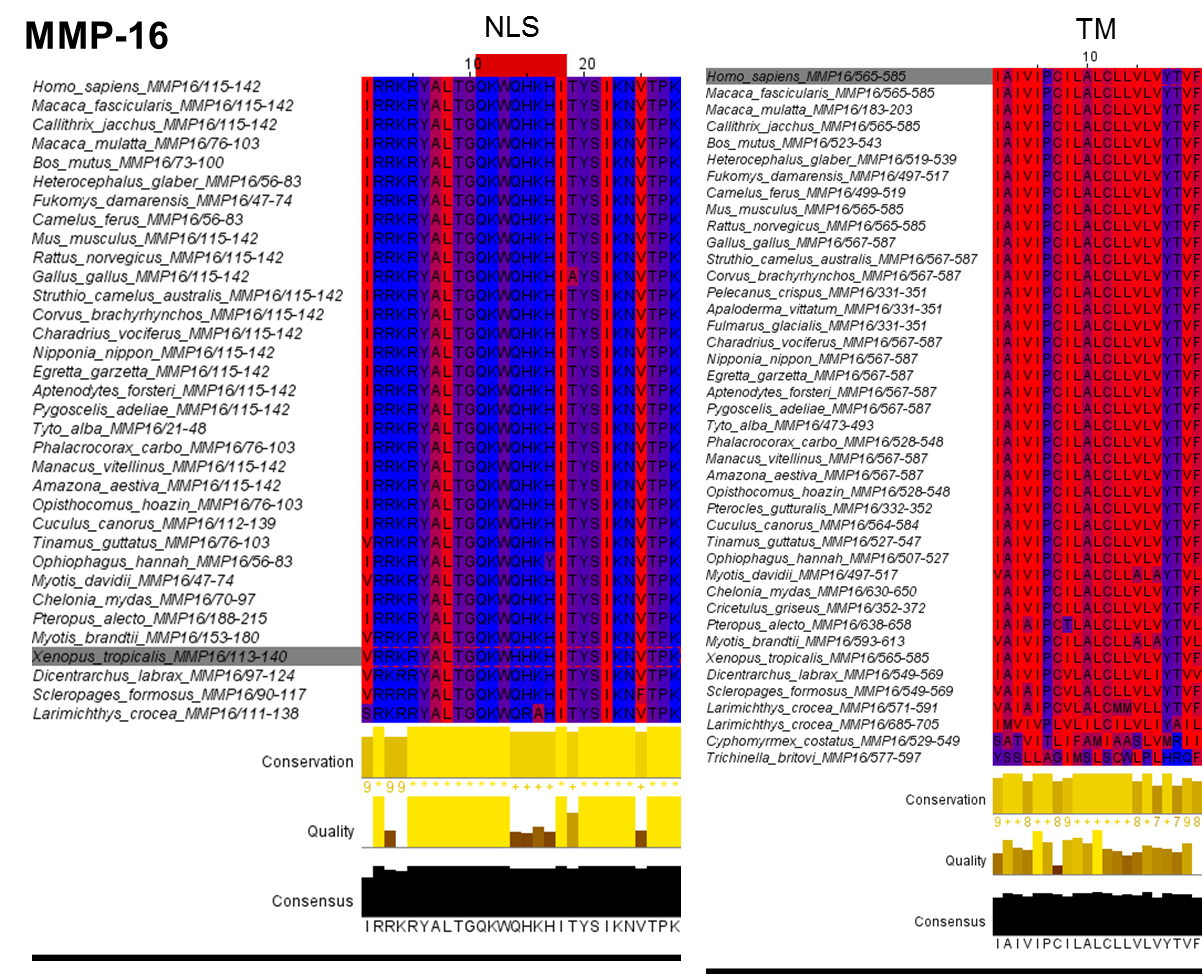


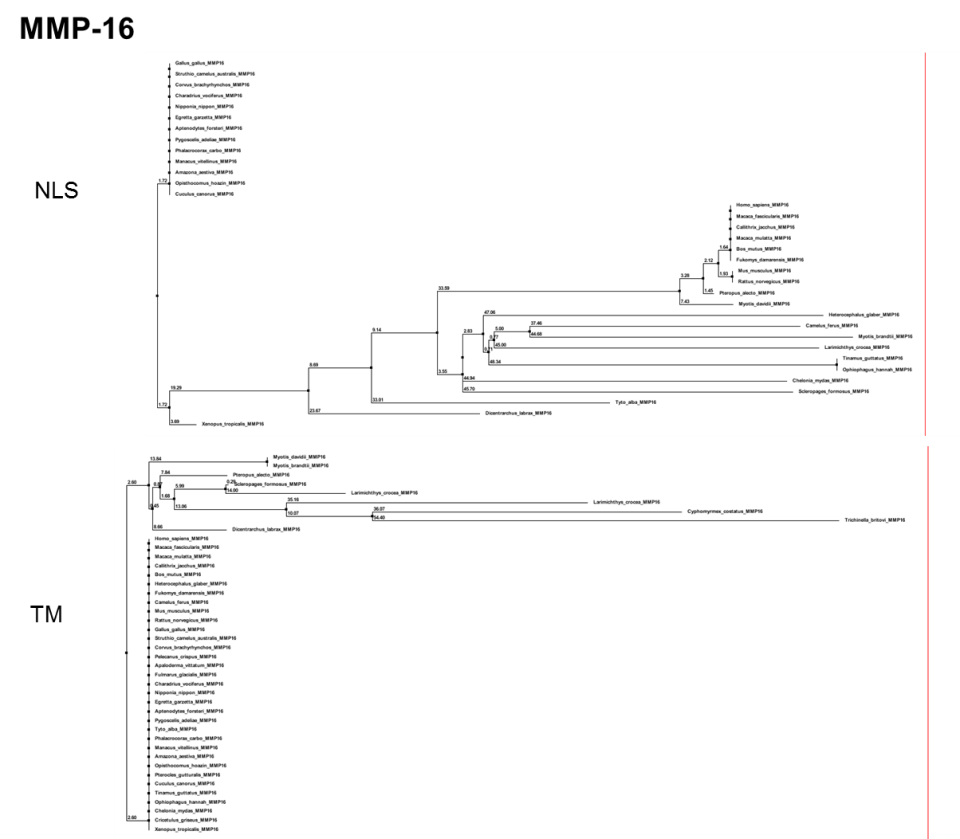


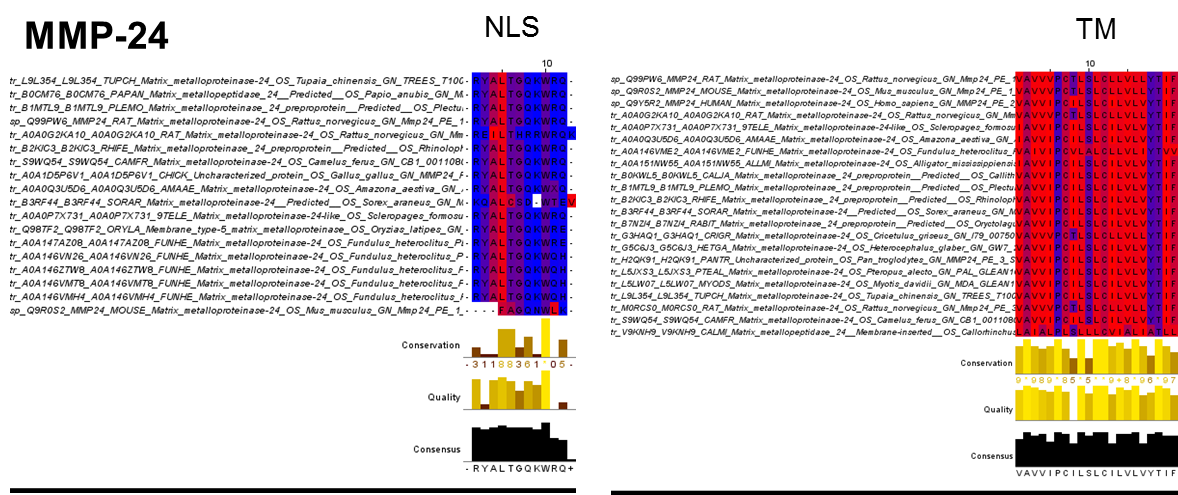


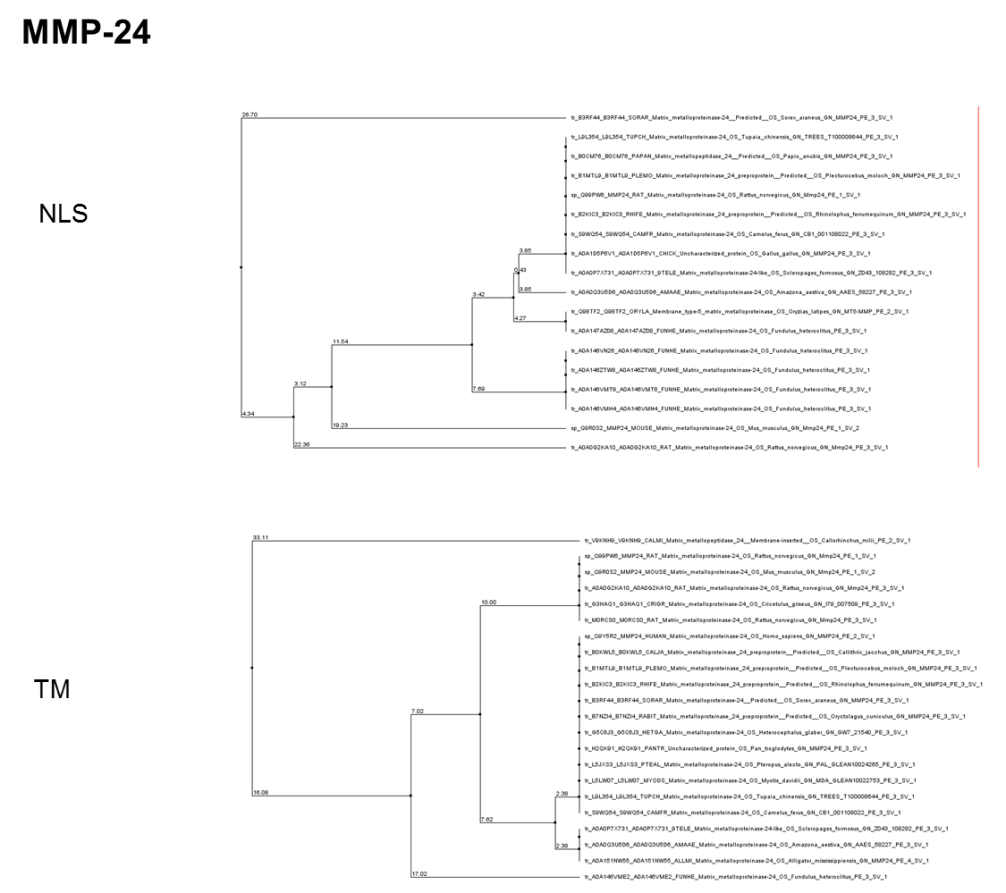


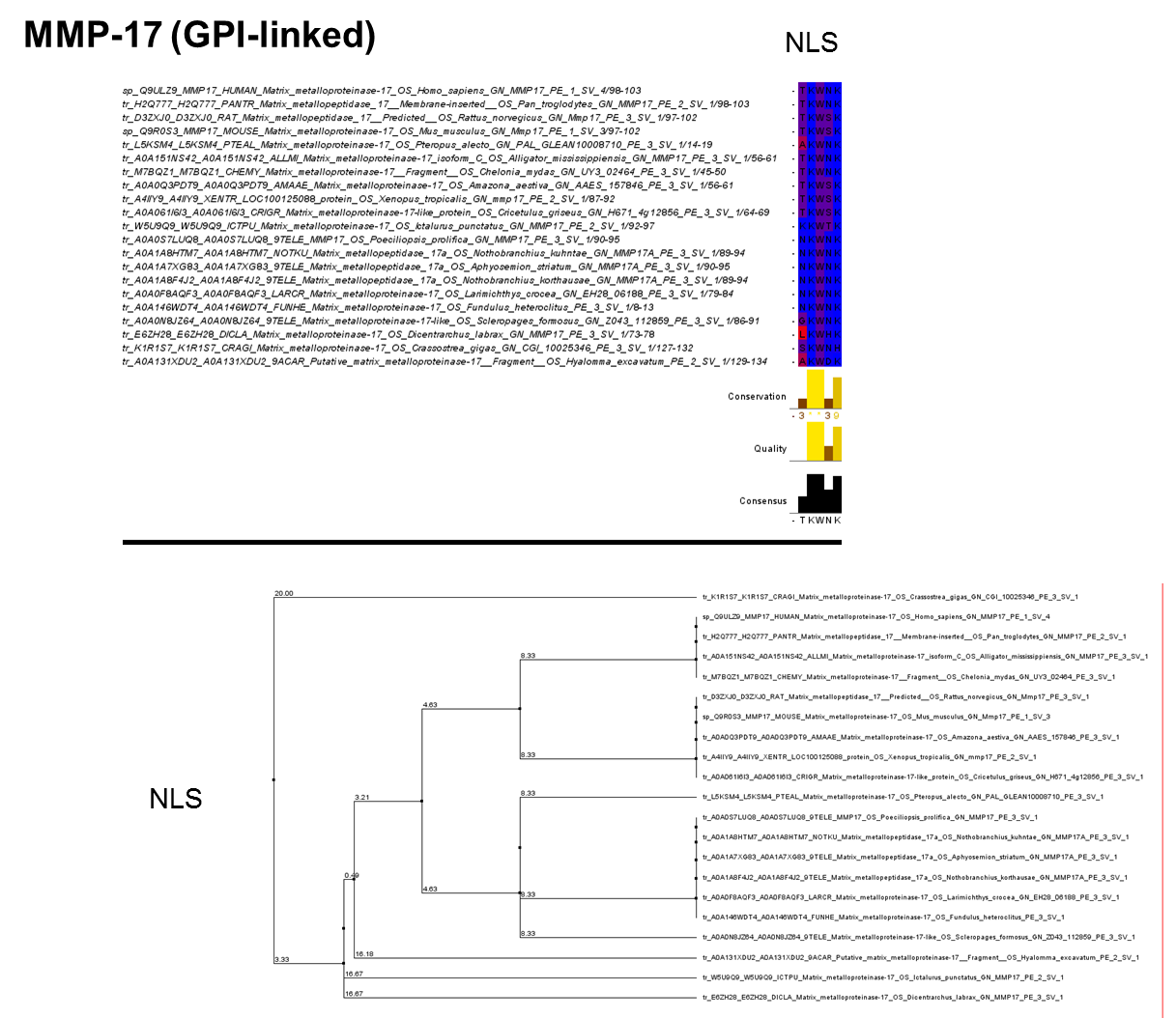


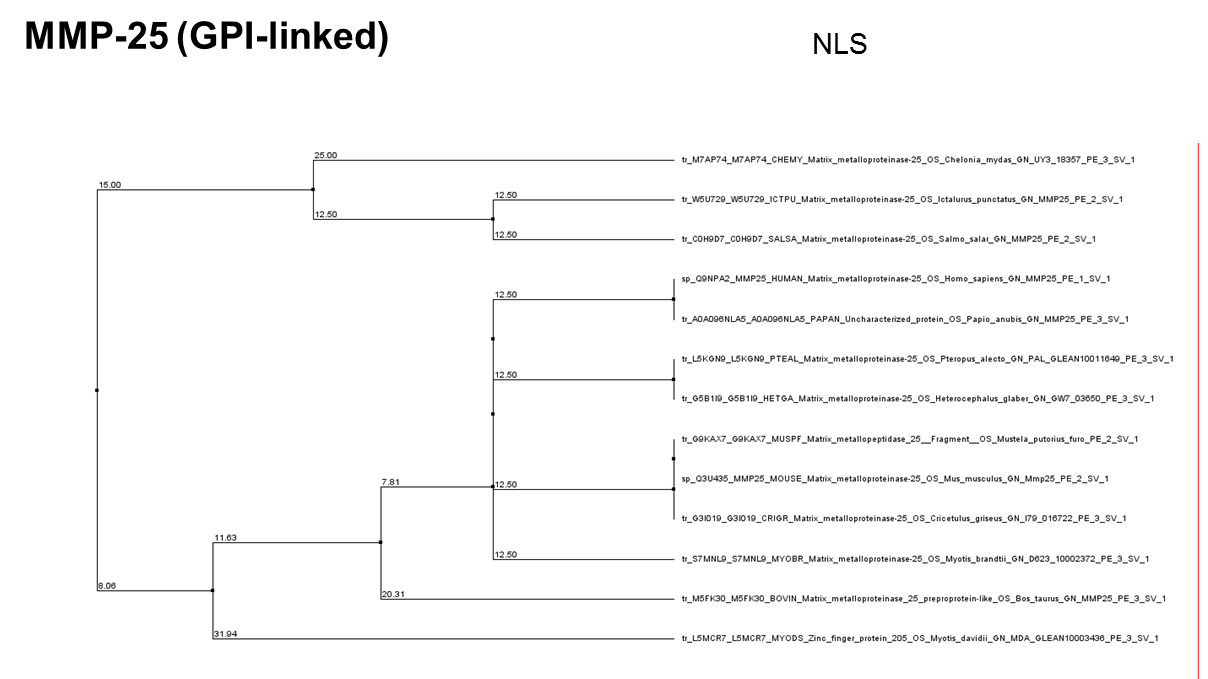

Supplement: Supplementary Information [file cddiscovery201736-s1.docx]
